# Forced sex and its predictors among students attending university: a cross-sectional study

**DOI:** 10.1186/s13690-022-00823-4

**Published:** 2022-02-17

**Authors:** Shayesteh Jahanfar, Parivash Ahmadpour, Mojgan Mirghafourvand

**Affiliations:** 1 MPH Program, Department of Public Health and Community Medicine, Tufts University School of Medicine, Boston, USA; 2 Midwifery Department, Tabriz University of Medical Sciences, Tabriz, Iran; 3 Social Determinants of Health Research Center, Nursing and Midwifery Faculty, Tabriz University of Medical Sciences, Tabriz, Iran

**Keywords:** Forced sex, Knowledge, Sexual attitude

## Abstract

**Background:**

Forced sex is associated with negative psychological health outcomes. This study aimed to determine the prevalence of forced sex and its predictors.

**Methods:**

This cross-sectional study was performed on 800 students of a university in USA using a random sampling method. Reproductive health electronic questionnaire was used for data collection. Due to the sensitive nature of the questionnaires and for anonymity, Qualtrics software was used. To estimate the extent of the effect of each of the independent variables (knowledge, attitude, as well as socio-demographic characteristics) on the dependent variable (forced sex), multivariate logistic regression was used.

**Results:**

About one-fifth of students (16.9%) had experienced forced sex. The variables of gender, knowledge about sexually transmitted diseases (STD), and sexual attitude were among the predictors of forced sex. This kind of sexual relationship was more likely to occur in girls than in boys (OR = 2.94, 95%CI: 1.20 to 1.71). Further, the chance of forced sex significantly increased with growing knowledge of STD (OR = 1.41, 95%CI: 1.61 to 1.71), and sexual attitude (OR = 1.23, 95%CI: 1.04 to 1.21).

**Conclusion:**

Considering the impact of gender, knowledge about STD, and sexual attitude on forced sex, educational interventions among the youth especially girls are required to provide complete and proper information about sexual and reproductive health and rights and correct the sexual attitudes of the youth.

## Background

Biological, psychological, and social changes of adolescence and youth period are the determining factors of health or disease in subsequent years of life [1]. For many teenagers, these changes occur without any adverse outcomes. Nevertheless, adopting risky behaviors in some adolescents may affect their sexual health [2, 3]. Social sciences scientists have used the term “forced sex” to refer to the interactions in which a person is pressured or forced to commit a sexual act against their will [4]. According to the World Health Organization (2018), sexual violence against children includes “completed sexual relation without consent or attempt for doing sexual relationship which even may not lead to a relationship (such as promiscuous activities or sexual harassment); committing rape against a person who is unable to give consent or refuse it” [5]. Sexual violence against youth is a very complex phenomenon. The agents of the creation of forced sex change during childhood, adolescence, and adulthood; younger children are primarily victimized by the family members, while the youth are victimized usually by acquaintances, intimate partners, foreigners, and those in authority. The incidence of sexual violence grows progressively from around 8 years of age, with the maximum rate of incidence of sexual violence for infants being the age range of 14–17 years [6, 7].

The precise prevalence of unwanted sexual experiences for adolescents is unknown. This gap of knowledge can be due to ambiguity in determining the nature of the experience due to the over-extensive and nonspecific nature of the questions posed in this regard [8]. Although this type of information is collected systematically among the samples of the national representatives of the adolescents (for example, a question has been considered about forced sex from 1999 in the national survey of the adolescent’s risky behaviors), due to shame, denial, self-blame or fear of violation, cases of violence may not be reported [9]. Further, cultural and religious norms, social structures, the school environment, and economic factors may also affect these issues [10]. According to studies, the prevalence of forced sex is higher among teenage girls than in boys, but teenage boys also report sexual violence [11]. In the US, researchers estimate that 6.1% of children experience sexual victimization at the age of 17 and younger. The prevalence of sexual abuse among girls and boys is 26.0 and 5.1%, respectively. Forced sexual initiation (FSI) has been reported to be 5–46% among women in low- and middle-income countries of Africa [12].

A study reported that there is a relationship between forced sex in teenagers and factors such as gender, negative self-concept, and suicidal ideation [13]. Forced sex has also been associated with attitude factors in adults such as low self-efficacy as well as sexual relationship associated behaviors, including a large number of past friendship partners [14, 15]. The risk of these factors is closely associated with socio-ecological factors, including social, cultural, religious, familial, educational, political, and ideological factors affecting lifestyle [14].

FSI can have adverse effects on both children and the youth. Various researchers have documented the high prevalence of forced sex, as well as its harmful effects and consequences in the US [16–18]. Sexual violence is associated with adverse psychological health, such as depression, and committing suicide. According to the results of a meta-analysis, sexual abuse increases the probability of depression and suicide commitment by 2.7 and 4.1 times, respectively. Also, the prevalence of associated PTSD and major depressive disorder is almost 2.5 times as large compared to the individuals who have not experienced sexual violence [16]. Apart from psychological effects, there are two other important serious concerns, including unwanted pregnancy and sexually transmitted infections (STIs) [19, 20]. It is estimated that almost half of adolescents 15–24 years of age would contract STIs. Annually, out of every 20 adolescents, one is afflicted with a sexual bacterial infection, with the average age of this disease showing a descending trend [21]. In recent years, the prevalence of human papillomavirus (HPV), *Neisseria gonorrhoeae*, HIV, and *chlamydia trachomatis* infection is growing, especially in younger individuals [22]. All of these sexual health problems will be more prevalent through sexual relations during adolescence, which in turn is reinforced with having several sexual partners, forced sex, unprotected sexual relationships, or not using condoms. FSI is associated with different types of high-risk sexual behaviors such as transactional sex, having numerous sexual partners, and not using condoms [23]. The adolescents and young adults experiencing FSI have reported not using condoms in their first and last sexual relations more than those who have not experienced FSI [24]. In women with an experience of FSI, the probability of contracting STI and HIV is higher compared to women without FSI experience. The young women who have experienced FSI will most probably experience the next forced sex, which is associated with the violence of an intimate partner [25].

Sexual health knowledge is the necessary prerequisite for sexual health and constitutes an inseparable part of human behavior and decision-making, especially when there is the ability to take informed protective measures. In addition, in sexual and reproductive rights, education as a vital protective factor has attracted attention among adolescents [26]. There is robust evidence suggesting that sex education based on the curriculum at school leads to increased awareness of sexual risk and threats, as well as knowledge about risk mitigation strategies, causing commitment to safer sexual relations [27]. Due to the wide social variety of comprehensive behavioral interventions targeting adolescents, consideration of the social context is required [28]. Attempts for correcting social norms are also needed to support change, preserve behavioral change, and to tackle structural factors involved in high-risk sexual behavior. The prerequisite of this type of intervention is establishing the status of forced sex and its associated factors, so that proper intervention could be designed and implemented in this regard [27]. Thus, the present study was performed to support filling this important gap to determine the prevalence of forced sex and its predictors.

## Methods/design

The STROBE reporting guidelines were followed in the reporting of this study.

### Study design and sampling

This was a cross-sectional survey among students attending university in Michigan, in the United States of America. The university has a population of approximately 27,000 students on its main campus, with students coming from various places across the globe. A sampling frame for the study was obtained from the Registrar’s Office. Students received an invitational email, and those who consent to participate in the study received an electronic reproductive health questionnaire containing questions on their sexual behavior. This study used a random sampling technique to send email invitations to CMU students in three rounds.

A calculated sample size (*n* = 800) was estimated using the prevalence of “being forced to have sexual intercourse” of 3%., with an expectant increase to 30%, 1.96 Z value, 5% of precision, a power of 80, and 10% non-respondent rate.

### Study population

The inclusion sampling criteria were CMU students who were registered as a student and were willing to participate in the study, while the exclusion criterion was a refusal to participate in this study. We did not include samples from the school of Health Sciences.

### Study questions

1. What is the distribution of sexual coercion among university students?
2. What is the relationship between forced sex and socio-demographic characteristics?
3. What is the relationship between forced sex and knowledge of contraception/Knowledge of STD/knowledge of condom use?
4. What is the relationship between forced sex and sex education?
5. What is the relationship between forced sex and risky sexual behavior?
6. What is the relationship between forced sex and sexual attitude?

### Data collection and analysis

We used a self-administered structured questionnaire derived from World Health Organization illustrative questionnaire [29]. The questionnaires were sent through Qualtrics online software when the survey links were sent via emails to the participants. Because of the sensitive nature of the questionnaire, anonymity was a priority in this research. The software facilitated the anonymous status of the students. Qualtrics complies with the EU-U.S. Privacy Shield Framework and the Swiss Privacy Shield framework. It retains the American Arbitration Association/International Centre for Dispute Resolution for disputes.

The questionnaire was adopted by the researchers and modified to reflect cultural sensitivities. The change was minor, and no language translation was necessary. We first sent the questionnaire to 10 students and two faculty members to check the content validity. The data from these ten students were excluded from the analysis. The original questionnaire sought to assess the knowledge, attitudes, and practice of students of CMU about reproductive health. Part of this study was about the sources of sexual health information and has been published elsewhere [30]. The current manuscript focuses on forced sexual coercion among students (the outcome variable).

First, we asked, “Some young people are forced to have sexual intercourse against their will by a stranger, a relative, or an older person. Has this ever happened to you?” (Yes/No). This variable was considered as the dependent variable. Another question related to sexual coercion was, “Did you or the sexual partner do anything to avoid pregnancy on these occasions? If Yes Always or sometimes?”.

We also asked questions about unsolicited touches: Some young people/females are touched on the breast or some other part of the body when they do not want to be, by a stranger, a relative, or an older person. Has this ever happened to you? (Yes/No).

Second, we studied the association between socio-demographic characteristics and forced sex. Third, we analyzed the relationship between forced sex and other potential confounders: level of knowledge (STD, condom, contraception), sexual attitude, and relationship score.

#### Knowledge of STD

Knowledge of sexual infectious diseases, including HIV/AIDS/ and sexually transmitted diseases, was also assessed using 10 questions from the same survey. These questions were inclusive of the following items: Q1. “Have you heard of HIV or AIDS?” (Yes = 1/No = 0), Q2. “Were you ever concerned that you might catch HIV/AIDS or another STD from your partner?” (Yes = 1/No = 0), Q3. “If you were concerned, were you very concerned or somewhat concerned?” (Very concern/somewhat concern = 1, not concerned = 0), Q4. “Did you do anything to reduce the risk of infection?” (Yes = 1/No = 0), Q5. “What did you do to reduce the risk of infection?” (Used condom = 1, took medication = 0), Q6. “People can take a simple test to find out whether they have HIV.” (True = 1, False = 0), Q7. “It is possible to cure AIDS.” (True = 1, False = 0), Q7. “A person with HIV always looks emaciated or unhealthy in some ways.” (Yes = 1/No = 0), Q8. “I haven’t had sex because I am afraid of getting HIV/AIDS or another STI.” (True = 1, False = 0), and Q9. “Condoms are an effective way of protecting against HIV/AIDS.” (True = 1, False = 0). These nine questions were recoded into correct and incorrect answers, and the total score of knowledge was computed by adding these scores together. The detailed data is not shown here.

#### Knowledge of condom (Section 9)

The total score of knowledge about condoms was estimated by adding 15 questions. The average score of knowledge was 12.25 ± 1.39, with the minimum 2 and maximum 15. The distribution of the score is normal. Please note that I coded disagree = 1 (reverse coding) and disagree = 0 (usual coding) and don’t know = 2, and agree = 1(usual coding) and agree = 0 (reverse coding). These questions are as follows: Q1. “Have you or a partner ever used a condom?” (Yes = 1/No = 0). Q2: “Have you ever experienced a condom that split or broke during intercourse?” (Yes = 1/No = 0). Q3: “Have you ever seen a condom?” (Yes = 1/No = 0). Q4: “Condoms are an effective method of preventing pregnancy?” (Agree = 1/ Don’t know/not sure = 2/ Disagree = 3). Q5: “Condoms can be used more than once?” (Agree = 1/ Don’t know/not sure = 2/ Disagree = 3). Q6: “A girl can suggest to her boyfriend that he use a condom?” (Agree = 1/ Don’t know/not sure = 2/ Disagree = 3). Q7: “A boy can suggest to his girlfriend that he use a condom?” (Agree = 1/ Don’t know/not sure = 2/ Disagree = 3). Q8: “Condoms are an effective way of protecting against HIV/AIDS?” (Agree = 1/ Don’t know/not sure = 2/ Disagree = 3). Q9: “Condoms are suitable for casual relationships?” (Agree = 1/ Don’t know/not sure = 2/ Disagree = 3). Q10: “Condoms are suitable for steady, loving relationships?” Q11: “It would be too embarrassing for someone like me to buy or obtain condoms?” (Agree = 1/ Don’t know/not sure = 2/ Disagree = 3). Q12: “If a girl suggested using condoms to her partner, it would mean that she didn’t trust him?” (Agree = 1/ Don’t know/not sure = 2/ Disagree = 3). Q13: “Condoms reduce sexual pleasure?” (Agree = 1/ Don’t know/not sure = 2/ Disagree = 3). Q14: “Condoms can slip off the man and disappear inside the woman’s bod?” (Agree = 1/ Don’t know/not sure = 2/ Disagree = 3). Q15: “If unmarried couples want to have sexual intercourse before marriage, they should use condoms.” (Agree = 1/ Don’t know/not sure = 2/ Disagree = 3).

#### Knowledge of contraception (Section 7)

Fifteen questions were used for this section. Q1: “Women can take a pill every day” (Yes (spot.) = 1/Yes (prompted) = 2 /No = 3). Q2: “Do you know any place or person where young people could obtain this method” (Yes = 1/No = 0). Q3: “Women can have an injection every 2 or every 3 months” (Yes (spont.) = 1/Yes (prompted) = 2 /No = 3). Q4: “Do you know any place or person where young people could obtain this method?” (Yes = 1/No = 0). Q5: “A man can put a rubber device on his penis before intercourse” (Yes (spont.) = 1/Yes (prompted) = 2 /No = 3). Q6: “Do you know any place or person where young people could obtain this method?” (Yes = 1/No = 0). Q7: “A woman can take pills soon after intercourse” (Yes (spont.) = 1/Yes (prompted) = 2 /No = 3). Q8: “Do you know any place or person where young people could obtain this method?” (Yes = 1/No = 0). Q9: “A man can pull out of a woman before climax” (Yes (spont.) = 1/Yes (prompted) = 2 /No = 3). Q9: “A couple can avoid sex on days when pregnancy is most likely to occur.” (Yes (spont.) = 1/Yes (prompted) = 2 /No = 3). Q10: “There are other methods of contraception that I have not mentioned. What other methods have you heard of?” (IUD = 1/ Implant = 2/ Jelly/foam = 3/ Female Sterilization = 4 / Male Sterilization = 5/ Other (SPECIFY) = 6). Q11: “Which method do you think is most suitable for young people?” (Pill = 1/ injection = 2/ Condom = 3/ Emerg. Pills = 4/ Withdrawal = 5/ Periodic. Ab =6 = Other = 7/ D.K = 8). Q12: “Which methods of contraception have you or a sexual partner ever used?” (Pill = 1/ injection = 2/ Condom = 3/ Emerg. Pills = 4/ Withdrawal = 5/ Periodic. Ab = 6/ Other = 7).

#### Sexual attitude (Section 10)

A total score of sexual behavior was computed by adding Q1 to Q23 as follows:

Q1. “I believe it’s all right for unmarried boys and girls to have dates” (Agree = 1, don’t know/not sure = 2, disagree = 3), Q2: “I believe it’s all right for boys and girls to kiss, hug and touch each other”. Q3: “I believe there is nothing wrong with unmarried boys and girls having sexual intercourse if they love each other.” Q4: “I think that sometimes a boy has to force a girl to have sex if he loves her”. Q5: “A boy will not respect a girl who agrees to have sex with him.” Q6: “Most girls who have sex before marriage regret it afterward.” Q7: “Most boys who have sex before marriage regret it afterward.” Q8: “A boy and a girl should have sex before they become engaged to see whether they are suited to each other.” Q9: “I believe that girls should remain virgins until they marry.” Q10: “I believe that boys should remain virgins until they marry.” Q11: “It is sometimes justifiable for a boy to hit his girlfriend.” Q12: “Most of my friends think that one-night stands are OK.” Q13: “It’s all right for boys and girls to have sex with each other provided that they use methods to stop pregnancy.” Q14: “Moat of my friends who have sex with someone use condoms regularly.” Q15: “I am confident that I can insist on condom use every time I have sex.” Q16: “I would never contemplate having an abortion myself or for my partner.” Q17: “It is mainly the woman’s responsibility to ensure that contraception is used regularly.” Q18: “I think that you should be in love with someone before having sex with them.” Q19:“ I feel that I know how to use a condom properly.” Q20: “Most of my friends would never contemplate having an abortion for themselves or their partner.” Q21: “Men need sex more frequently than do women” Q22: “Most of my friends believe that you should be in love before you have sex with someone” Q23: “I would refuse to have sex with someone who is not prepared to use a condom.”

#### Relationship score

The questions on sexual behavior consisted of the following: Q1. “How many girl/boyfriends have you had?” (More than median 3 = 1, 3 or less =0), Q2. “How old is your current girlfriend/boyfriend?” (Age difference was calculated using this variable and age of the participant (±3 years = 0, less than 3 or more than 3 = 1), Q3: “When you started your relationship, was your girlfriend/boyfriend single (coded 0) or married/divorced/ separated (coded 1)?” Q4. “When you started your relationship with your girlfriend/boyfriend, was she/he a fulltime student/working (coded 0) or neither (coded 1)?”, “When you started your relationship with your girlfriend/boyfriend, was she/he a full-time student, working or neither?” Q5. “Has the relationship ended?” (Yes = 1/No = 0), Q6. “How long was your relationship before ended? (10 month or shorter=1, 11 months or longer =0), Q7. “Who decided to end the relationship? (Both you and your girl/boyfriend = 0, one side = 1), Q8. “During the time you were/have been ‘dating’ your girlfriend/boyfriend, did you ‘date’/have you ‘dated’ anyone else?” (Yes = 1/No = 0), Q9. “How would you describe your relationship with your girlfriend/boyfriend? (Casual=1 or serious. towards marriage/engaged to be married=0), Q10. “How do you think your girlfriend/boyfriend would describe her /his relationship to you?” (Casual = 1 or serious/towards marriage/engaged to be married = 0).

### Data analysis

Statistical analysis of this data was done using IBM SPSS Statistics, version 26.0 released in 2019 (IBM Corp., Armonk, NY, USA). Descriptive analysis, bivariate, and logistic regression were performed to analyze this data. Regression analysis was conducted to find the predictive relationship between the forced sex and socio-demographic characteristics and level of knowledge, attitude, and relationship scores. As the total score of forced sex is a categorical variable, the odds ratio (OR) and the 95% confidence intervals (CI) were calculated. Missing data were less than 20% which is forgivable for analytical purposes.

### Ethical considerations

The protocol of the study was approved by the Ethics committee of the Institutional Review Board (IRB) at CMU (IRB: 1031916–4). We first sent an email to participants. In that invitation letter, the purpose of the study was explained. Students were asked to spend 20 min to fill up the questionnaire. Students were assured that the data would be confidential as no identification was collected from the students. The risk and benefits of participation in the study were explained to the participants. General publications and reports coming out of this study were sent to students for general education purposes. Students who participate in the study were eligible to receive a coupon for Pizza. Students were free to refuse participation or discontinuation of the study at any time point. They could also report any insensitivity in handling the research by researches to the IRB committee. Also, students were able to volunteer information to the researcher in order to get help.

## Results

Over 1100 students were approached at first step, all of whom were eligible to attend the study. Eligibility was confirmed for those who attended. Some students failed to respond to the complete questionnaire. These students were excluded from analysis. Overall, 20% missing data were found including those who did not fill up the questionnaire at all and those who partly filled up the questionnaire.

The mean (standard deviation) of the age of participants was 24.06 (9.61). More than two-thirds of students (68.2%) had an undergraduate academic level. The mean monthly income of the participants was $1000 or less. Most students (95.4%) were single, and most of them (90.5%) had not received any sexual education. About two-thirds of the participants (60.7%) had expressed that they are religious, and more than two-thirds (69.4%) of participants always used contraceptive methods. More than three-fourths of participants (87.3%) consumed alcohol, and less than one fifth (12.6%) smoked cigarettes. Finally, more than three-fourths of participants (77.9%) had visited bars/parties, and most students (93.3%) went to movies (Table 1).

**Table 1:** Socio-demographic characteristics of students attending university, 2019 (*n* = 800)

Variables^a^	N (%)
**Age (Mean ± SD)**	24.06 **±** 9.61
**Gender**	
Male	166 (20.7)
Female	634 (79.3)
**Education**	
Undergraduate	546 (68.2)
Graduate	254 (31.8)
**Work**	
Yes	520 (68.8)
No	236 (31.2)
**Income**	
$1000 or less	430 (66.7)
More than $1000	215 (33.3)
**Are you a religious person?**	
Yes	471 (60.7)
No	305 (39.3)
**Relationship status**	
Single	601 (95.4)
No single	29 (4.6)
**Practice-ever contraceptive use**	
Always	327 (69.4)
Never/sometimes	144 (30.6)
**Sex education**	
Yes	65 (9.5)
No	619 (90.5)
**Smoking**	
Yes	96 (12.6)
No	667 (87.4)
**Alcohol**	
Yes	662 (87.3)
No	96 (12.7)
**Visited bars/parties**	
Yes	597 (77.9)
No	169 (22.1)
**Went to movies**	
Yes	711 (93.3)
No	51 (6.7)
**Family source of information**	
Yes	262 (87.6)
No	37 (12.4)

^a^All variables except age, gender and education include missing cases

Close to one-fifth of students (16.9%) had experienced forced sex, out of whom, most (96.1%) were coerced by one person for the forced sex. The forced sex for 36.2% of participants had occurred through touching the breast or other parts of the body (Table 2).

**Table 2:** Distribution of forced sex among students attending university, 2019 (*n* = 800)

	N (%)
Some young people are forced to have sexual intercourse …. Has this ever happened to you?	
Yes	96 (16.9)
No	473 (83.1)
How many different strangers, relatives or older persons have forced you to have sex against your will?	
One	63 (96.1)
More than one	31 (3.9)
Some young people/females are touched on the breast or some other part …Has this ever happened to you?	
Yes	100 (36.2)
No	176 (63.8)
Would you say this has happened often, sometimes, or rarely?	
Often	56 (15.0)
Sometimes	68 (18.2)
Rarely	250 (66.8)

Based on the results of the bivariate tests, there was a significant relationship between gender and forced sex, which was more frequent in female students than in male students (*p* = 0.01). Also, there was a significant relationship between taking any contraceptive measures (p = 0.01) as well as being invited to visited bars/parties (*p* < 0.05) and forced sex. Invitation to the parties and the permanent use of any contraceptive method were more frequent in those who had forced sex. However, there was no significant relationship between the level of education, having occupation, income, marital status, religious beliefs, sexual education, alcohol consumption, and cigarette smoking, going to movies, as well as receiving or not receiving information from the family, and forced sex (*p* > 0.05). There was also a significant relationship between forced sex and knowledge of STD (*p* = 0.01) as well as sexual attitude (*p* = 0.01). However, no significant relationship was found between knowledge of condoms, contraceptive knowledge, as well as sexual behavior, and forced sex (*p*<0.05) (Table 3).

**Table 3:** The relationship between forced sex and socio-demographic characteristics of students attending university, 2019 (*n* = 800)

	Forced sex+ *N* = 95	Forced sex- *N* = 472	*P* values
**Age (Mean ± SD)**	23.52 ± 6.94	23.41 ± 6.92	0.89
**Gender**			
Male	10 (8.7)	105 (91.3)	0.01
Female	85 (18.8)	368 (81.2)	
**Education**			
Undergraduate	27 (16.2)	140 (83.8)	0.81
Graduate	69 (17.2)	333 (82.8)	
**Work**			
Yes	67 (18.3)	299 (81.7)	0.40
No	28 (15.1)	157 (84.9)	
**Income**			
$1000 or less	64 (20.0)	256 (80.0)	0.15
More than $1000	20 (14.3)	120 (85.7)	
**Are you a religious person?**			
Yes	63 (18.0)	287 (82.0)	0.42
No	33 (15.1)	186 (84.9)	
**Relationship status**			
Single	91 (18.6)	399 (81.4)	0.39
No single	2 (9.5)	19 (90.5)	
**Practice-ever contraceptive use**			
Always	49 (15.8)	262 (84.2)	0.01
Never/sometimes	36 (25.6)	103 (74.1)	
**Sex education**			
Yes	12 (21.1)	45 (78.9)	0.35
No	81 (16.1)	423 (83.9)	
**Smoking**			
Yes	12 (21.1)	45 (78.9)	0.35
No	81 (16.1)	423 (83.9)	
**Alcohol**			
Yes	85 (17.5)	400 (82.5)	0.18
No	9 (11.4)	70 (88.6)	
**Visited bars/parties**			
Yes	81 (18.6)	355 (81.4)	0.05
No	15 (11.4)	117 (88.6)	
**Went to movies**			
Yes	88 (16.7)	439 (83.3)	0.54
No	7 (17.1)	34 (82.9)	
**Family source of information**			
Yes	6 (20.7)	23 (79.3)	0.86
No	36 (19.3)	151 (80.7)	
Knowledge of STD	4.57 ± 1.83	3.98 ± 1.59	0.01
Knowledge of condom	12.24 ± 1.44	12.25 ± 1.38	0.96
Knowledge of contraception	16.63 ± 2.23	16.50 ± 2.36	0.67
Relationship behaviour	10.44 ± 1.33	9.85 ± 1.29	0.21
Sexual attitude	43.76 ± 3.64	42.19 ± 3.61	0.01

Based on the results obtained from the univariate logistic regression test, there was a significant relationship between forced sex and gender, the use of contraceptive methods, being invited to visit bars or parties, knowledge of STD, and sexual attitude (*p* < 0.05). On the other hand, based on the multivariate logistic regression model, the variables of gender, knowledge of STD, and sexual attitude were among the predictors of forced sex, where the chance of forced sex was higher in girls than in boys (OR = 2.94, 95% CI: 1.20 to 7.17, *P* = 0.01). Also, the chance of forced sex significantly increased with higher awareness of STD (OR = 1.41, 95% CI: 1.61 to 1.71) and sexual attitude (OR = 1.23, 95% CI: 1.04 to 1.21) (Table 4).

**Table 4:** Predictors of forced sex based on logistic regression among students attending university, 2019 (*n* = 800)

Variable	Unadjusted OR 95% CI	Adjusted OR 95% CI
**Age (Mean ± SD)**	1.00 (0.97–1.03)	
**Gender**		
Female	2.43 (1.22–4.84)	2.94 (1.20–7.17)
Male	1	
**Education**		–
Undergraduate	0.93 (0.57–1.51)	
Graduate	1	
**Work**		–
Yes	1.26 (0.78–2.03)	
No	1	
**Income**		–
$1000 or less	1.50 (0.86–2.59)	
More than $1000	1	
**Are you a religious person?**		–
Yes	1.24 (0.78–1.96)	
No	1	
**Relationship status**		–
Single	2.17 (0.49–9.47)	
No single	1	
**Practice-ever contraceptive use**		–
Always	0.54 (0.33–0.87)	
Never/sometimes	1	
**Sex education**		
Yes	1.39 (0.71–2.75)	
No	1	–
**Smoking**		–
Yes	0.56 (0.30–1.03)	
No	1	
**Alcohol**		–
Yes	0.61 (0.29–1.26)	
No	1	
**Visited bars/parties**		–
Yes	0.56 (0.31–1.01)	
No	1	
**Went to movies**		–
Yes	1.03 (0.44–2.31)	
No	1	
**Family source of information**		–
Yes	1.09 (0.42–2.88)	
No	1	
Knowledge of STD	1.24 (1.08–1.41)	1.41 (1.61–1.71)
Knowledge of condom	0.99 (0.82–1.19)	–
Knowledge of contraception	1.03 (0.91–1.15)	–
Relationship behaviour	1.45 (0.81–2.59)	–
Sexual attitude	1.13 (1.05–1.22)	1.23 (1.04–1.21)

## Discussion

The results obtained from the present study indicated that close to one-fifth of students had an experience of forced sex. The variables of gender, knowledge of STD, and sexual attitude were among the predictors of forced sex. The chance of such a sexual relation was higher in girls by 2.94 times compared to boys. Further, forced sex was more frequent in those with high knowledge of STD and sexual attitudes.

In the present study, the prevalence of forced sex among participants was 16.9%. In a study by Tsai et al. [31], the prevalence of forced sex among women in Botswana and Swaziland was 10.3 and 11.4%, respectively. The prevalence of this relationship for the men of these two countries was 3.9 and 5.0%, respectively. The difference in the statistics of the general prevalence of forced sex can due to the notable ambiguity in the definition of such behavior [32]. Any attempts for forced sex can be in the form of threats of violence, physical force or poisoning, or subtler techniques such as emotional manipulation. In an intimate relationship where the two persons love each other, we may expect that forced sex sometimes occurs with subtler manipulation rather than explicit threats and the use of physical force [33]. The definition by Waldner et al. [34] for forced sex where “sexual behavior may range from touching and kissing to sexual intercourse” implies this issue.

Gender was one of the predictors of forced sex in the present study. The higher prevalence of forced sex among girls compared to boys has been reported in other studies [35, 36]. According to WHO (2002) [37], up to one-third of adult girls have reported forced sex in their first sexual relationship. In Peru, 47% of adolescent girls had reported a history of forced sex [38]. Among women who had registered for HIV tests and HIV counseling in the US, the prevalence of rape throughout their lifetime was reported 43% [39].

In the present study, in addition to gender, knowledge of STD, and sexual attitude were among the other predictors of forced sex. Various factors have been reported as the elements of women’s sexual behavior, including poverty, sociocultural factors, economic factors, educational factors [40], and knowledge of reproductive health [41]. In a Sierra Leone study, women’s understanding of exposure to the risk of AIDS had a strong correlation with the probability of incidence of forced sex. This can be due to increased women’s understanding of the status of risk of contracting HIV and STD from their partner, their increasing unwillingness to commit sexual relationships, and the provocation of forced behaviors by their spouse. A woman’s understanding of the status of HIV and STD in their partner is associated with refusing sexual relations and coercion [42].

Understanding factors (e.g., knowledge, attitude, and behavioral skills) affecting the sexual behaviors of the youth and adolescence is very important. This is because it has a great influence on sexual decision-making, where educational programs and behavior can considerably improve these factors. The knowledge constituents the basis of human action [43]. This is a principal concept in many psychological theories and is usually used as a theoretical basis for having an effective sexual relationship as well as STD/HIV educational programs. The knowledge may also affect the behavior indirectly by influencing the values, attitudes, understanding norms, and even self-efficacy. Nevertheless, having knowledge and information does not necessarily guarantee the behavior, since knowledge alone is not sufficient. Behavior attitude is so important that the person’s tendency to that behavior is determined by its desirability or undesirability [44].

The sources that possibly affect the level of knowledge and sexual attitude of the youth are different. The parent-adolescent relationship may be one of the useful strategies to improve healthy sexual and reproductive behavior. Talking to adolescents about issues related to sexual relationships, including contraception, avoiding pregnancy, methods of HIV prevention as well as preventing another STIs is a positive method for reducing the sexual risk among teenagers and adopting a safer sexual behavior [26]. Nevertheless, the communication established by adults regarding sexual and reproductive health (SRH) is affected by social norms as well as taboos associated with gender, religious and cultural beliefs, as well as sexual desires [45].

### Strengths and limitations

The large sample size and random selection of participants were among the strengths points. Although the participants were chosen from the same university, the students at this university come from various parts of the world. Hence, its results can be generalized to other countries as well. In this study, to prevent response bias, the research tool was completed using Qualtrics online software and with no name. The limitation was the cross-sectional nature of this study, where the indicated relationships may not exactly reflect causal relations. Thus, it is proposed to conduct studies with a more robust design to identify the factors associated with forced sex. As most adolescents and youth are interested in social networks and websites [28], it is suggested to use these communication channels to present proper information about sexual and reproductive health.

## Conclusions

The results of the present study indicated that the variables of gender, knowledge of STD, and sexual attitude are among the predictors of forced sex. Thus, considering the pattern of gender, implementing proper behavioral, educational interventions among the youth, especially girls for reducing forced sex, as well as presenting complete and proper information about SRH, are required to enhance the awareness and correct the attitudes of the youth.

## Data Availability

Datasets used and analyzed during this study are available from the corresponding author on reasonable request.

## Abbreviations

US: United States
FSI: Forced sexual initiation
PTSD: Post Traumatic Stress Disorder
STIs: Sexually transmitted infections
HPV: Human Papillomavirus
HIV: Human Immunodeficiency Virus
IRB: Institutional Review Board
AIDS: Acquired Immunodeficiency Syndrome
OR: Odds Ratio
CI: Confidence Intervals
WHO: World Health Organization
SRH: Sexual and Reproductive Health
